# Impact of Films: Changes in Young People’s Attitudes after Watching a Movie

**DOI:** 10.3390/bs10050086

**Published:** 2020-05-02

**Authors:** Tina Kubrak

**Affiliations:** Laboratory of Speech Psychology and Psycholinguistics, Institute of Psychology, Russian Academy of Sciences, 13, Yaroslavskaya, 129366 Moscow, Russia; kubrak.tina@gmail.com; Tel.: +7-926-378-2946

**Keywords:** psychology of cinema, influence of mass media, impact of films, attitudes, attitudes towards elderly people

## Abstract

Nowadays films occupy a significant portion of the media products consumed by people. In Russia, cinema is being considered as a means of individual and social transformation, which makes a contribution to the formation of the Russian audience’s outlook, including their attitudes towards topical social issues. At the same time, the question of the effectiveness of films’ impact remains an open question in psychological science. According to the empirical orientation of our approach to the study of mass media influence, our goal was to obtain new data on the positive impact of films based on specific experimental research. The task was to identify changes in the attitudes of young people, as the most active viewers, towards topical social issues after watching a specifically selected film. Using a psychosemantic technique that included 25 scales designed to identify attitudes towards elderly people, respondents evaluated their various characteristics before and after watching the film. Using a number of characteristics related to the motivational, emotional and cognitive spheres, significant changes were revealed. At the same time, significant differences were found in assessments of the elderly between undergraduate students and postgraduate students. After watching the film, postgraduate students’ attitudes towards elderly people changed in a positive way, while undergraduate students’ negative assessments only worsened. The revealed opposite trends can be explained by individual differences of respondents, which include age, educational status as an indicator of individual psychological characteristics, the experience of interaction with elderly people and, as a result, attitudes towards elderly people at the time before watching the movie. The finding that previous attitudes mediate the impact of the film complements the ideas of the contribution of individual differences to media effects. Most of the changes detected immediately after watching the movie did not remain over time. A single movie viewing did not have a lasting effect on viewers’ attitudes, and it suggests the further task of identifying mechanisms of the sustainability of changes.

## 1. Introduction

With the development of information technology, a person’s immersion in the field of mass media is steadily increasing. A significant portion of consumed media products is occupied by cinema. According to sociological surveys, going to the cinema is the most popular way of spending leisure time in Russia today (http://www.fond-kino.ru/news/kto-ty-rossijskij-kinozritel/); the audiences of cinemas are growing, the core of which are 18-24 years olds, as well as the frequency of visits—every tenth Russian goes to the cinema several times a month (https://wciom.ru/index.php?id=236&uid=1785; https://wciom.ru/fileadmin/file/reports_conferences/2018/2018-04-03_kino.pdf; http://www.fond-kino.ru/news/portret-kinoauditorii-rezultaty-monitoringa-za-i-kvartal-2019-goda/), the opportunities and frequency of Internet viewings is expanding, while interest in TV shows is also increasing. The importance of the role that cinema plays in Russia is also confirmed by the close attention currently being paid to the development of the cinema industry: the priority topics of state financing are defined (e.g., “Law and order: the heroes of modern society in the fight against crime terror, extremism and corruption”, “On the continuity of military generations, on the successors of military traditions”, "Images, patterns of behavior and creative motivation of our contemporary—a man of labor, in the military or a scientist"), while state programs are being launched to open new cinema theatres in small towns. Сinema becomes a “tool for broadcasting state ideology to the masses” (according to S. Zizek [1]), and is also being considered as a “means of individual and social transformation” (according to T. Kashani [2]) [3]. As a result, films are expected to form beliefs, influence opinions and change attitudes, including towards topical social issues.

However, the question of the efficiency of films remains open in psychology. In general, this is a key issue for mass communication research: how much emotion, cognition and behavior are changed under the influence of mass media [4,5]. There are various concepts about this: from “theories of a minimal effect” to “theories of a strong effect” [6]. Thus, for example, cultivation theory considers that mass communication contributes to the assimilation of commonly accepted values, norms and forms of behavior [7]; and a meta-analysis of studies leads to the conclusion that there is a relationship between the broadcast mass media image of reality and people’s attitudes towards it [8,9]. Despite criticisms, cultivation theory is currently being developed [10,11,12,13]. On the other hand, supporters of the opposite viewpoint point out the weak effects of mass communication, caused, for example, by the fact that people are becoming more and more subject of their mass media activity as a result of a wider variety of sources of information now and expanding their choices [14,15].

It seems difficult to identify a single mechanism of mass media impact on the human psyche and behavior and to obtain an unambiguous answer to the question about its efficiency [6]. This is due to the interconnection of various factors that mediate the influence of mass media (personal experience, realistic content, depth of identification with heroes, personality traits, etc.) [16,17,18], as well as those factors that constantly impact persons besides those in the media. Therefore, our thoughts and ideas about this issue are largely based on empirical research data, and are not limited to one theory [19].

When referring to research of cinema, we can find data on the diverse effects of film exposure. It should be noted that the effectiveness of the impact is determined by what it is directed at: it is more difficult to change human behavior than to influence opinions or attitudes [4,6]. In this regard, there is still a debatable problem on the influence of the media on the aggressive behavior of people [20]. This research focuses on the potential of pro-social, "humanistic" impact of films and their effectiveness in solving topical social issues. The studies reveal the influence of films on people’s beliefs and opinions, stereotypes and attitudes. Movies can have a significant impact on gender and ethnic stereotypes [21,22], change attitudes towards certain groups of people and cause newly formed opinions on various issues. For example, HIV films contributed to sympathy to people living with HIV [4], TV series with transgender characters contributed to positive attitudes towards transgender persons [23]; the portrayal of mental disorders in movies had an effect on people’s knowledge about and attitudes toward the mentally ill [24,25]. Also, viewing an empathy-arousing film about immigrants induced more positive attitudes toward them [26], and watching a movie offering a positive depiction of gay men reduced homophobia [27]. Other films influenced people’s attitude towards smoking and their intentions to quit [28,29], while a series with a positive donation message helped viewers to make decisions about their own donation [30]. It has been shown that emotional involvement in viewing, evaluated using surveys drawing on theories of social learning and social representations, increases the effectiveness of influence [30]; immersion in narrative, that correlates with the need for cognition, and is characterized by a shift of focus from the real world to the depicted one, explains the power of impact within the framework of transportation theory [31,32]. 

Cinema can change people’s opinions on specific issues without affecting more stable constructs: for example, the film “JFK” dedicated to the Kennedy assassination influenced judgments about the causes of this crime, but generally did not change the political beliefs of the audience [33]; at the same time, the movies “Argo” and “Zero Dark Thirty” changed viewers’ opinions about the U.S. government that reflected in an improvement in sentiments about this government and its institutions [34]. Movies create images of other countries and stimulate interest in them. For example, European films shaped young viewers’ ideas about other European countries—such results were obtained in a study of the role of films and series in the daily life of young Germans through interviews and focus groups [35]. Another study showed that whether the movies were violent, scary or happy, the more the viewers were immersed in the stories, the more favorable impressions they had of the places featured in them [36].

Various positive effects of films on children and adolescents were revealed. Dramatic films taught teenagers about social interaction with the opposite sex and adults [37], had a positive impact on their self-concept [38], and, as shown by experiments, increased ethnic tolerance [39]; humanistically oriented movies improved skills of children in communicating with peers, increased their desire to help and understand others [40,41].

One of the prime examples of positive impact is Cli-fi movies, which clearly show what we can expect in the near future, and offers ways to think about what can be done to avoid the darkest predictions. Thus, after watching the film “The Day After Tomorrow” (2004), viewers recognized their responsibility for the Earth’s ecology and the need to change consumer attitudes towards nature [42]. In general, the screening of films on climate issues increases the number of online requests and media discussions on these issues [43].

It should be noted that when analyzing the impact of films, conclusions about their effectiveness are the result of different methodological approaches, which have varying advantages and limitations. Content analysis reveals the images, attitudes, stereotypes broadcast by films (e.g., stereotypical portrayals of India [44], or images of scientists and current scientific ideas [45]) on large data sets; however, questions remain about effectiveness, strength and sustainability of the impact on the audience. The influence of films can be investigated through a survey of viewers; based on this, conclusions are drawn about the links between a person’s attitudes and his/her viewer experience, such as in the study of gender attitudes and their correlations with teen movie-viewing habits [21]. In experimental studies, exposure effects are detected using pre- and post-film questionnaires; however, the time interval between testing and a film screening, such as a few weeks before viewing the film or a several days after [26,27,29], can lead to distortion of the results that are caused by the influence on the viewers’ attitudes of other factors besides the film; moreover, usually it is not investigated whether new attitudes are retained over time. Often the effects of films are analyzed in experimental conditions where participants watch only short cut scenes from existing films [24], which limits the extrapolation of the results.

According to the empirical orientation of our approach, the goal was to obtain new data on the positive impact of films based on a specific experimental study. The task was to identify changes in young people’s attitudes towards topical social issues after watching a specifically selected film. Participants had to watch the full version of an existing fiction film. They were tested just before and immediately after watching the movie in order to avoid the influence of other variables on viewers’ attitudes. Repeated testing (two weeks after the first viewing) was intended to reveal the sustainability of the changes caused by the film.

In the process of developing the design of the work, it was specified what attitudes would be studied. The choice was determined, first of all, by the social relevance of the topic, but outside the focus of the media in order to reduce the impact of other media sources, and on the other hand, by the availability of a suitable film. Important topics as ethnic stereotypes, attitudes toward people with disabilities, etc. were considered. However, the choice of the topics had to be restricted for various reasons. For example, identifying attitudes toward certain professions (e.g., engineer), whose prestige has significantly declined in Russia in recent decades, was difficult due to the lack of relevant films popularizing them. At the same time, despite the availability of humanistically oriented films dedicated to people with disabilities, the identification of changes in attitudes to them was complicated by the need to take into account additional factors caused by increased attention to the topic and active discussion in various media, which could distort the influence of a film.

Given the limitations and opportunities for the implementation of research tasks, the subject of this study the attitudes towards elderly people. At present, attention to the topic concerning elderly people is growing in Russia, but there is still a prevalence of negative stereotypes [46]. A characteristic manifestation of age discrimination against the elderly—ageism—is a biased attitude towards them, especially among young people, as well as a low assessment of their intellectual abilities, activity and "usefulness" for society. 

Studies show that the mass media have a significant impact on negative attitudes towards the elderly [47]: children have already demonstrated the same stereotypes of the elderly that were depicted in the media [48], while young people at large viewed the elderly in general as ineffective, dependent, lonely, poor, angry and disabled, which corresponded to the negative representations of elderly people in the most popular teen movies that cultivated their stereotypes [49]. Research of TV films from the 1980s–1990s revealed the stereotypes of elderly people as being social outsiders [50], but at the same time a display of positive prejudice contributed to an increase in tolerance towards them within society. 

Improving the attitudes of young people towards elderly people is an important social and educational task, the solution of which involves the use of diverse opportunities. Various social projects can be implemented for this purpose, for example, "friendly visitor" types of programs in which young people visit the elderly [51,52], but also mass media, including films, which have a high potential for impact [53,54]. It was found that watching documentary films had a positive effect on both knowledge about aging and attitudes towards the elderly [55]; these films significantly improved empathy towards elderly people among university students [56].

We suggested that fiction movies, popular especially among young people, could contribute to changing existing biased attitudes towards elderly people. Based on this, the hypothesis states that there is a connection between watching a positive film about the elderly and changes in young people’s attitudes towards them in a positive way. 

## 2. Methods

### 2.1. Participants

A total of 70 individuals participated in this study. Group one contained 40 students of The State Academic University for Humanities (25% male and 75% female). The average age was 19 (M = 19, standard deviation SD = 2.4). Group two consisted of 30 postgraduate students from Russian Academy of Sciences (47% male and 53 % female). The average age was 24 (M = 24, standard deviation SD = 1.6).

All subjects gave their informed consent for inclusion before they participated in the study. The study was conducted in accordance with the Declaration of Helsinki and was approved by the local ethics committee (Review Board).

### 2.2. Materials

#### 2.2.1. Film

The film—“The Best Exotic Marigold Hotel” (2011), the main characters of which were elderly people, was chosen to be shown to the respondents (https://www.imdb.com/title/tt1412386/). Prior to this, a qualitative analysis of empirical material revealed the impact of this film on the attitudes towards elderly people among Russian viewers of different ages. Their reviews on the film, taken from Internet resources devoted to cinema, indicated cognitive effects, expressed in positive changes of ideas about the elderly; the film was perceived quite optimistically and gave hope [19]. It was supposed that the movie that humorously shows various situations happening to the elderly heroes would also affect the opinion of young people about elderly people, as it allowed to look at them from new viewpoints, to see that age is not an obstacle to having a full life, and even, conversely, open up new prospects.

#### 2.2.2. Measures

To achieve the goal of the study, a psychosemantic approach is used, which is the most appropriate for studying a person’s attitudes towards various objects of reality by reconstructing individual meanings [57]. This approach allows us to determine the differences in evaluations of the same object (caused by mass media as well), made by different groups of respondents at different times. For example, changes in the stereotypes of viewers were revealed in relation to representatives of another nation (Russians about the Japanese) during viewing of a TV show [57]. In this work, the psychosemantic technique was used, developed specifically to identify attitudes towards the elderly (based on the Kelly’s Repertoire lattice method) [46]. The technique included 25 7-point scales, according to which respondents rated elderly people. For comparative analysis, the modern youth were evaluated by participants with the same scales.

The respondents also noted the frequency of watching movies ("every day"/"several times a week"/"several times a month"/"several times a year and less"), and evaluated the level of enjoying the film shown ("did not like"/"rather did not like than liked"/"rather liked than disliked"/"liked").

### 2.3. Procedure

The study was conducted in three stages: the respondents filled out the psychosemantic test before watching the film, then immediately after viewing and again in 2 weeks. During stage 3, only group one participated in the study.

The respondents did not see the film before participating in the study.

### 2.4. Statistical Methods

In accordance with the data characteristics, non-parametric comparative methods were used. To determine the differences in the assessments before and after watching the movie, the Wilcoxon signed-rank test was used. To determine the differences in the assessments between different groups of respondents, the Mann-Whitney U test was used. The IBM SPSS Statistics 20 statistical software package was used for data processing.

## 3. Results and Discussion 

As a result of the preliminary data analysis of the group one (students), significant differences were obtained in the assessments given by them to the elderly before and immediately after watching the movie (Wilcoxon signed-rank test, *p* < 0.05). However, the analysis of the combined sample (students and postgraduate students) did not reveal such significant differences. Therefore, it was decided to compare the assessments of these two groups of respondents. It appeared that the evaluation of elderly people differed among students and postgraduate students before the film was shown (18 of 25 scales, Mann—Whitney test, *p* < 0.05). This result could be explained by the individual differences of the participants (students and postgraduates), which led to the necessity to correct the hypothesis and form additional research tasks, including the comparison of groups. Further analysis was carried out separately for each group of respondents, but not for the united group.

Significant differences shown by respondents of the group one before and immediately after watching the film (students) were found in 12 out of the 25 scales (Table 1).

The group of students revealed changes associated with ideas about activities. The respondents saw the elderly as having less initiative, and being purposeless and weak. Moreover, they defined elderly people’s way of life as more passive, having no desire for knowledge or for living a full life. The results immediately after watching the film demonstrated that the audience perceived the elderly as being those who strived less to learn new things and perceived them to be less positive and more limited in their interests. Also, the changes of assessments related to the emotional sphere were discovered. The elderly were characterized as even more unrestrained and conflict-prone with a tendency towards depression and showing no emotions.

Comparative analysis of assessments of elderly people before and after watching the film, given by respondents of the group two (postgraduate students), showed significant differences on 14 of the 25 scales (Table 2). Postgraduate students evaluated the elderly, unlike students, more positively after watching the film. Changes on 9 common scales (purposeless - purposeful, cheerful - prone to depression, passive - initiative, conflict - peaceful, traditional - modern, etc.) for students and postgraduate students turned out to be of different directions. After watching the film, the elderly seemed to be more purposeful, active and successful, responsible and with a good sense of humor. There were changes in assessments of the emotional sphere (more cheerful, peaceful) and cognitive (more intelligent) in references to novelty and life in general (the strive to learn new things, the desire for a full life). 

Thus, the data revealed changes in attitude towards the elderly people after watching the film. According to a number of their characteristics related to motivational aspects—regulatory, emotional and cognitive spheres—significant changes were revealed, but the tendency of these changes was unexpected. After group one (students) watched the film, a tendency of worsening assessments was found. It was also determined that before the film, students described the elderly more negatively as being less intelligent and interesting, more conflict prone, angry and aggressive than young people (Wilcoxon signed-rank test, *p* < 0.01). This generally negative attitude can be explained by a special view of quite young people on the "old age". But why, despite the attempt of the filmmakers to make the image of the elderly positive enough, did the film fail to change students’ attitude? Instead, it made the image of elderly persons even less attractive. Meanwhile, there was an opposite trend in group two (postgraduate students). Their assessments of elderly people after watching the film changed for the better. The postgraduate students, unlike undergraduate students, had already demonstrated a more "adequate" view on the elderly before watching the film. Despite a number of negative assessments, the elderly were seen by them as smart and striving for a full life, sociable and interesting. 

Comparison of the two groups of respondents confirmed significant differences between students and postgraduate students in the evaluation of the elderly after watching the film (Table 3). The assessments given by undergraduate students and postgraduates differed significantly on 21 out of 25 scales.

The opposite tendencies found in assessments after watching the film could be explained by differences in individual characteristics of respondents, which were not initially considered in our study as factors mediating the impact of the film: age of respondents (more subtle differentiation), educational status, as an indicator of individual psychological characteristics and experiences of interactions with elderly people. The suggestion of differences between students and postgraduates by personality is consistent with the results of other studies [58], and is indirectly confirmed by the fact that only about 1 out of 40 students become postgraduate students (data for Russia). In our study, differences between students and postgraduate students already manifested in differences in their attitudes towards the elderly before watching the film. 

Then the film, which showed some negative aspects of life for elderly people (loneliness, needlessness, diseases, fears, physical limitations “comic” behavior), despite the optimistic ending, could strengthen the negative attitudes of very young people (students) towards the elderly, whose images might not yet be fully formed. On the other hand, postgraduate students might have a more complex view on elderly people, because of age and more diverse interactions with the elderly, for example, in scientific work. In this case, their perceptions of the film could be focused on its positive ideas, strengthening their previously formed positive image of an elderly person. In addition, postgraduate students, who have chosen the scientific career path, most likely have a high level of analytical skills that contributed to more complex perceptions of the world and a deep assessment of the phenomena that could affect their attitudes towards the older generation and the interpretation of their images in the movies. At the same time, the characteristics of the film itself, as well as the cultural differences between its creators and viewers, might cause additional negative impacts on students’ perceptions. Comedy, as a genre, could have an opposite effect. Younger people perceived the desire of older characters to give their lives new meanings in their own way and they saw a futility in these attempts. Respondents with more experience could be more tolerant to the specifics of the genre, and their perception of the film was more complicated and implemented in a broader context.

Thus, comparison of the results of the analysis for both groups of respondents suggests that the different changes in viewers’ attitudes towards objects of reality that occur after watching a movie can be explained by differences in the attitudes before watching the film. This effect can also be explained by the degree of identification with the characters [31,59,60], which is influenced by the previous attitudes of the viewers. For example, a study of the impact of films on attitudes towards migrants showed that greater identification with the characters induced more positive attitudes toward immigration, but only when previous prejudice was low or moderate [26]. In this regard, the various effects of the film on students and postgraduate students could be caused by the different degrees of their identification with the characters of the film, despite the fact that a large difference in age with the characters could complicate this process for all participants in the study. The conclusion that previous attitudes mediate the impact of the film complements the ideas of the contribution of individual differences to media effects [61]. In addition, this conclusion has practical value: in order to achieve the desired impact of films, it is necessary to identify the viewers’ individual attitudes before a screening. 

At the third stage of the study, it was examined whether changes remained over time. Two weeks after watching the movie, respondents (group one) re-took the test. 

Significant differences were found only on 4 scales (strives to a full life - lost the meaning of life, craving for spirituality - limited interests, quickly tired – high in stamina, traditional - modern) (Table 4). The continuing changes in the characteristics related to the inferiority and limitations of elderly people’s lives may indicate the most striking and memorable moments in the film that had the greatest impact on viewers. The assessments of the other characteristics did not differ significantly from those that were identified before watching the film. That leads to the conclusion that a single movie viewing, in general, did not have a lasting effect on the viewers’ attitudes toward the elderly. Most of the changes discovered immediately after watching the movie did not remain over time. Studying the mechanisms of the formation of sustainable changes is a task for future research. One of the directions of such research could be to investigate the influence of additional cognitive processing (e.g., discussion after watching the movie) on the viewers’ attitudes towards objects and the sustainability of changes over time.

The correlation between the gender of the respondents and changes in attitudes after watching the film was determined by comparing the assessments separately for males and females in each group. As a result, in group one, women were found to have significant differences in ratings on 13 scales, and men in three, two of which were common (no desire to learn anything - the desire to learn new skills, traditional - modern, Wilcoxon signed-rank test, *p* < 0.05). The data showed greater changes in the attitudes among women than among men after watching the film. At the same time, a comparison of the male and female participants in the group two did not reveal such results. The analysis found an equal number of significant differences in assessments (on 10 scales) before and after watching the film (Wilcoxon signed-rank test, *p* < 0.05). Thus, it can be assumed that gender had a lower impact on changes in attitudes after watching a film than other individual characteristics of respondents. 

The data on the frequency of watching movies was obtained: 56% of respondents watch movies several times a week and more often, 44%—several times a month and less often. However, there were no differences between these viewers in the assessments before and after watching the film (Mann-Whitney U test, *p* < 0.05). The degree of general interest in cinema did not affect the change of viewers’ attitudes after watching the film.

It was not possible to determine the connection between liking the film and the changes in attitudes, since the differentiation of respondents by this factor was not found. Only six young people noted they did not like the film, while the others gave it a positive evaluation.

The study has limitations caused due to an assumption of no significant differences between students and postgraduates in the effectiveness of the film’s impact on them. The revealed differences between undergraduate students and postgraduate students led to the initial sample of young people being divided into two samples with smaller sizes already used during the research. In addition, for the same reason, some variables that could more accurately demonstrate the differences between students and postgraduate students and explain the effects of the film were not considered. The respondents’ attitudes before watching the film were taken into account, as well as additional factors on the impact effectiveness, such as the degree of general interest in cinema and liking of the viewed film, which could presumably increase its impacts. But a deeper study, for example, of the processes of identifying viewers with the film characters, probably linked to the viewers’ attitudes before watching, could reinforce these findings.

## 4. Conclusions

As a result of the study, changes in the viewers’ attitudes after watching the film were identified. Young people changed their assessments of regulatory, cognitive and emotional characteristics of the elderly people after watching a film about the elderly. At the same time, significant differences were found between students and postgraduate students in their assessments of the elderly. After watching the film, students’ negative attitudes towards elderly people got worse, while postgraduate students’ assessments, on the contrary, changed for the better. The revealed opposite trends can be explained by individual differences between the respondents, which include age, educational status as an indicator of individual psychological characteristics, experience of interaction with elderly people and, as a result, attitudes towards elderly people at the time before watching the film. Most of the changes in the viewers’ attitudes detected immediately after watching the movie did not remain over time. 

In general, the study confirms the potential for a positive impact, as in the case of improving the postgraduates’ attitudes, but at the same time demonstrates the need to take into account the individual differences of viewers to achieve desired results. In particular, differences in attitudes before watching a movie are probably causes of differences in the effectiveness of the film’s impact. The initially negative attitude towards elderly people among students could contribute to the negative influence of the film on them. The obtained results form the basis of further research and pose the important questions: clarifying the contribution of individual differences to the effectiveness of the impact, forecasting the positive influence of movies on different groups of people and determining the mechanisms of the sustainability of changes.

## Figures and Tables

**Table 1:** Changes in assessments of the elderly people after watching the film (students).

Scales	Mean before Watching the Film	Mean after Watching the Film	Z	Asymp. Sig. (2-tailed)
emotional - unemotional*	1.10	1.68	-2.787b	0.005
purposeless - purposeful	−0.17	−1.18	−3.187c	0.001
active life position - having no life goal*	−0.02	1.21	−3.121b	0.002
no desire to learn anything - the desire to learn a new skill	0.22	−1.03	−3.899c	0.000
cheerful - prone to depression*	0.40	1.02	−2.053b	0.040
passive - initiative	0.28	−0.88	−3.325c	0.001
unrestrained – self-contained	−0.03	−0.65	−2.038c	0.042
conflict - peaceful	−0.28	−0.80	−2.281c	0.023
strives for a full life - lost the meaning of life*	0.35	1.35	−3.451b	0.001
craving for spirituality - limited interests*	0.68	1.67	−2.791b	0.005
quickly tired - having a high stamina	1.68	0.37	−3.420c	0.001
new is not perceived to be good - a modern view on new things	1.35	−0.62	−4.894c	0.000

Wilcoxon signed-rank test. Only the significant differences are represented: b—based on negative ranks, c—based on positive ranks. * inversive scales: a higher rating means a more negative attitude

**Table 2:** Changes in assessments of elderly people after watching the film (postgraduates).

Scales	Mean before Watching the Film	Mean after Watching the Film	Z	Asymp. Sig. (2-tailed)
smart - stupid	−0.40	−1.10	−3.383b	0.001
purposeless - purposeful	−0.17	0.60	−3.036c	0.002
active life position - having no life goal*	−0.67	−0.47	−2.347b	0.019
no desire to learn anything - the desire to learn new skills	−1.07	0.23	−3.795c	0.000
cheerful - prone to depression*	0.33	−0.97	−3.795b	0.002
passive - initiative	−0.53	0.37	−2.673c	0.008
takes responsibility - blames others for problems	0.40	−0.53	−3.077b	0.002
conflict - peaceful	−0.40	0.33	−2.729c	0.006
strives for a full life - lost the meaning of life*	−0.20	−1.07	−2.361b	0.018
quickly tired – high stamina	−1.73	−0.47	−3.376c	0.001
considered someone else’s opinions - imposing their own opinions*	1.30	−0.10	−4.065b	0.000
have achieved in life what they wanted - many things in life could not do *	0.77	−0.20	−3.050b	0.002
traditional - modern	−1.37	−0.17	−3.493c	0.000
with a sense of humor - without a sense of humor	−0.57	−1.10	−2.430b	0.015

Wilcoxon signed-rank test. The significant differences are only represented: b—based on positive ranks, c—based on negative ranks. * inversive scales: higher rating means more negative attitude.

**Table 3:** Comparison of groups of undergraduate students and postgraduates by assessments after watching the film.

Scales	Mean Rank Group one	Mean Rank Group two	Z	Asymp. Sig. (2-tailed)
smart - stupid	48.83	17.73	−6.430	0.000
emotional - unemotional	47.59	19.38	−5.31	0.000
always comes to the rescue of others - only cares for oneself	46.44	20.92	−5.290	0.000
purposeless – purposeful	25.81	46.95	−4.413	0.000
An active life position - having no life goal*	43.85	23.50	−4.270	0.000
no desire to learn anything - the desire to learn new skills	27.24	45.08	−3.759	0.000
cheerful - prone to depression*	45.84	21.72	−4.984	0.000
passive – has initiative	28.35	45.03	−3.479	0.001
interesting to chat - it is hard to find a topic for communication with them	45.21	21.73	−4.888	0.000
takes responsibility - blames others for problems	42.64	25.98	−3.439	0.001
life experience is out of date - wise	27.49	46.18	−3.869	0.000
persistent - does not complete goals	48.23	18.53	−6.176	0.000
conflict - peaceful	25.99	43.43	−2.865	0.004
strives for a full life - lost the meaning of life	46.45	20.90	−5.281	0.000
craving for spirituality - limited interests	48.53	18.13	−6.262	0.000
balanced - aggressive	43.58	24.73	−3.903	0.000
fast tired - stamina	39.78	29.80	−2.067	0.039
with a sense of humor - without a sense of humor	48.06	18.75	−6.089	0.000
undecisive - decisive	24.46	50.22	−5.358	0.000
communicative - closed	48.06	18.75	−6.046	0.000
cruel - kind	25.95	48.23	−4.632	0.000

Mann-Whitney U test. The significant differences are only represented.

**Table 4:** Changes in assessments of the elderly people 2 weeks after watching the film (students).

Scales	Mean before Watching the Film	Mean in 2 Weeks after Watching the Film	Z	Asymp. Sig. (2-tailed)
emotional - unemotional*	1.10	1.14		
purposeless - purposeful	−0.17	−0.43		
active life position - having no life goal*	−0.02	0.23		
no desire to learn anything – the desire to learn a new skill	0.22	−0.14		
cheerful - prone to depression*	0.40	0.77		
passive - initiative	0.28	−0.23		
unrestrained - self-contained	−0.03	0.06		
conflict - peaceful	−0.28	−0.11		
strives for a full life - lost the meaning of life*	0.35	1.00	−2.050b	0.040
craving for spirituality - limited interests*	0.68	1.31	−2.037b	0.042
quickly tired - high stamina	1.68	1.03	−2.096c	0.036
traditional - modern	1.35	0.63	−2.850c	0.004

Wilcoxon signed-rank test. Only the significant differences are represented: b—based on negative ranks, c—based on positive ranks. * inversive scales: a higher rating means a more negative attitude.
